# Patient-reported outcomes with golimumab in patients with rheumatoid arthritis, psoriatic arthritis, and ankylosing spondylitis: non-interventional study GO-NICE in Germany

**DOI:** 10.1007/s00296-018-4180-4

**Published:** 2018-11-10

**Authors:** Klaus Krüger, Gerd R. Burmester, Siegfried Wassenberg, Martin Bohl-Bühler, Matthias H. Thomas

**Affiliations:** 1 Rheumatologisches Praxiszentrum, Munich, Germany; 2 Department of Rheumatology and Clinical Immunology, Charité-Universitätsmedizin, Berlin, Germany; 3 Rheumazentrum, Ratingen, Germany; 4 Rheumahaus Potsdam GbR, Potsdam, Germany; 5 Medical Affairs, MSD Sharp & Dohme GmbH, Lindenplatz 1, 85540 Haar, Germany

**Keywords:** Golimumab, Rheumatoid arthritis, Psoriatic arthritis, Ankylosing spondylitis, Patient reported outcomes, Non-interventional study

## Abstract

The TNF inhibitor golimumab (GLM) is a treatment option in patients with rheumatoid arthritis (RA), psoriatic arthritis (PsA), and ankylosing spondylitis (AS). The GO-NICE study assessed patient-reported outcomes (PRO) in patients newly treated with monthly GLM 50 mg subcutaneously (SC) under real-life conditions in Germany. A prospective non-interventional study with 24-month observation per patient was conducted at 158 sites. Available for analysis were 1,458 patients, 474 with rheumatoid arthritis (RA: 54.9 ± 13.4 years, 72.8% females, 60.4% biologic-naïve), 501 with psoriatic arthritis (PsA: 50.5 ± 12.1 years, 54.1% females; 47.5% biologic-naïve), and 483 with ankylosing spondylitis (AS: 43.6 ± 12.3 years, 66.5% males; 58.4% biologic-naïve). A total of 664 patients completed follow-up to month 24. An improvement of QoL by EuroQoL EQ-5D-3L was seen after 6 months and was maintained over 24 months. The patients’ health state today (EQ visual analog scale) improved statistically significantly (*p* < 0.0001 vs. BL) from 51.0 at baseline (BL) to 63.4 (RA), from 48.4 to 64.3 (PsA) and from 46.8 to 66.5 (AS). Functional ability (FFbH) improved significantly (*p* < 0.003 vs. BL) from BL 68.2 to 76.1 points (RA), from 69.0 to 76.8 points (PsA), and from 69.0 to 78.5 points (AS). The mean FACIT-Fatigue score increased significantly (*p* < 0.0001 vs. BL) from BL 32.4 to 38.3 points (RA), from 30.0 to 35.9 points (PsA), and from 29.9 to 37.9 points after 24 months (AS); *p* < 0.0001 vs. BL each. On treatment with GLM SC once monthly, significant improvements in patient-reported QoL parameters were noted in a very similar manner in all three diseases.

**Trial registration** ClinTrials.gov Identifier: NCT01313858. Registered March 14, 2011; https://clinicaltrials.gov/ct2/show/record/NCT01313858.

## Background

Patient-related outcomes (PRO) are defined as reports provided by patients about their own health, quality of life, or functional status associated with the health care or treatment they have received [1]. The importance of including the patient perspective by assessing PROs in clinical trials has been emphasized, among others, by major international health policy and regulatory authorities [2, 3].

Rheumatic diseases in general lead to pain, joint damage, fatigue, and reduced activities of daily living and mobility [4]. Therefore, quality of life (QoL) and other PRO measures have received high attention in this indication [5–7]. The European League Against Rheumatism (EULAR) emphasizes that patient perspectives and priorities in treatment decisions are an overarching principle of care for rheumatoid arthritis (RA) patients [8].

Rheumatoid arthritis, (RA) and the spondyloarthropathies psoriatic arthritis (PsA) and ankylosing spondylitis (AS)—as three common immune-mediated rheumatic diseases—have different phenotypes but share important pathophysiologic mechanisms. Also, they are tackled with similar treatment approaches with anti-inflammatory medications, including non-steroidal anti-inflammatory drugs, glucocorticoids, disease-modifying anti-rheumatic drugs (DMARDs), and biologics including tumour necrosis factor inhibitors (TNFi). The newer biological agents have been reported to have a beneficial impact on health-related QoL and productivity in RA [9].

Golimumab (GLM) is one of the newer, second-generation TNF inhibitors [10]. It is injected once a month subcutaneously, permitting self-injection and thus greater flexibility and convenience for patients. The efficacy and safety of GLM has been shown in these indications in a number of large-scale randomised controlled trials (RCT) [11–14], and their open-label 5-year extensions [15–18]. These studies have entered selected patients in terms of disease characteristics, comorbidities and concomitant medications, and have been performed nearly a decade ago [19].

Data on the effect of GLM on the patient-reported outcomes (PROs) quality of life (QoL), fatigue, and functionality in daily clinical practice are lacking.

In a previous analysis, the effectiveness and safety of GLM 50 mg SC once monthly was shown in patients with RA, PsA, and AS in a real-life setting in Germany. In that study, Disease Activity Score 28-joint count erythrocyte sedimentation rate (DAS28-ESR) decreased from 5.0 to 2.9 after 24 months (*p* < 0.0001) in patients with RA, and Bath Ankylosing Spondylitis Disease Index score decreased from 5.1 to 2.4 (*p* < 0.0001) in patients with AS. Response rate calculated in patients with PsA by modified Psoriatic Arthritis Response Criteria was 67.9% after 24 months. Thus, during the 24-month observation periods substantial improvement in disease activity (DAS28 and BASDAI), response (PsARC) was seen early at 3 months and was maintained throughout the 24-month observation period in line with the previous clinical studies [20].

Thus, the present analysis of the pragmatic GO-NICE study aims to describe the effects of GLM treatment from the patient perspective using PROs in the indications RA, PsA, and AS, under daily practice conditions.

## Methods

### Study design

GO-NICE is an open-label, multicenter, prospective observational study, which was performed between 2010 and 2015 at 158 German sites, mostly by rheumatologists (92.4% of the physicians). Details on the study design, patient characteristics, the clinical outcomes, and safety data have been reported earlier [20].

Patients were managed according to the treating physician’s clinical judgment and without any protocol-defined therapeutic or diagnostic procedures. In addition, no inclusion and exclusion criteria were specified in contrast to interventional studies as observational studies are open to all patients eligible to receive treatment according to the prescribing information [21].

### Patients

Adult patients were eligible if they met the inclusion criteria: diagnosis of RA, PsA or AS; absence of any contraindication for GLM; patient consent for participation; no prior treatment with GLM and indication for use of GLM applied subcutaneously (SC) with an auto-injector at a dosage of 50 mg as specified in the product labelling [22]. There were no explicit exclusion criteria to avoid patient selection bias. All patients were treated with GLM 50 mg SC according to the Summary of Product Characteristics (SPC). Patients were evaluated prior to the first administration of GLM and in 3-monthly intervals thereafter over a 2-year period.

### Physician assessments

Physician Global Assessment (PhGA) is a non-disease-specific evaluation of participants’ overall health status assessed on a visual analogue scale (VAS) ranging from “0” (free of complaints) to “10” (strong discomfort). The closer the score is to 0, the better the health status. PhGA was assessed in all three groups by the treating physician on a VAS ranging from 0 = free of complaints to 10 = strong discomfort.

### PRO instruments and assessments

#### EuroQoL

General healthcare evaluation was done with the EuroQoL five-dimensional questionnaire (EQ-5D-3L). It consists of five domains (mobility, self-care, usual activities, pain/discomfort, and anxiety/depression); each is divided into three levels of perceived problems (“no, some or extreme problems”). In addition, patients rate their current health on a 20-cm vertical visual analogue scale (VAS) scored from 0 to 100 reflecting the continuum from the best imaginable to the worst imaginable health state. The EQ-5D-3L is validated for the German population [23].

#### Hannover functional ability questionnaire

The FFbH is a participant questionnaire assessing disability/functional impairment. Ability to perform 18 activities of daily living are scored on a 3-point scale (2 = Yes, 1 = Yes but with effort, and 0 = No or with assistance) and summed. Remaining functional capacity is calculated as the percent of the maximum number of score points (FFbH = (Attained score × 100)/(2 × *n*) where n is the number of completed responses) with range from 0 = total loss of functional capacity to 100 = maximal functional capacity. Increase from baseline in FFbH score signifies improvement. The FFbH is similar to Health Assessment Questionnaire (HAQ) but is more widely used in Germany, and both show a high degree of correspondence, as a validation study in RA patients in Germany has shown [24].

#### Fatigue

Fatigue was assessed with the Functional Assessment of Chronic Illness Therapy Fatigue (FACIT-F) questionnaire, originally developed for cancer, in which patients score (0–52) tiredness, weakness, and difficulty with usual activities because of fatigue (increased score indicates reduced fatigue) [25]. The questionnaire was validated in a PsA cohort in a Canadian study, showing high internal consistency, test–retest reliability, and criterion and construct validity [26]. Also, it was responsive to change in a trial of adalimumab in PsA [27]. Reliability and validity of the German translation of the FACIT-Fatigue Scale were confirmed recently [28].

### Data management and statistics

Investigators or their staff used an online electronic data capture system to enter clinical parameters into the database. Patients provided PRO data on paper forms, which were entered into the database by trained staff of a contract research organisation. In a subset of centres, study data were compared with the patient files (source data verification).

Analyses were performed in an exploratory manner using descriptive statistical methods. All effectiveness analyses were conducted for the evaluable patients (who had the baseline (BL) assessment and at least one additional visit) and the completer patients (who had the BL assessment and the visit at month 24), grouped by indication [20]. For continuous variables, the number of patients with non-missing data, mean, SD, minimum, 25% quartile, median, 75% quartile and maximum were calculated. For ordinal and categorical variables, frequencies were calculated based on all observations with non-missing data for this variable. Incomplete datasets were included in the analysis. Missing GLM treatment start date was replaced by date of visit 1. There was no imputation of missing values for any endpoint. Changes in variables by time were calculated with least square means, standard error of the man, and 95% confidence intervals. P-values were calculated with chi square or Wilcoxon tests, for PhGA with signed rank test. No sensitivity analyses were performed. PRO data were analysed based on the instructions of the respective tool developers.

## Results

### Patient disposition

**Fig. 1:**
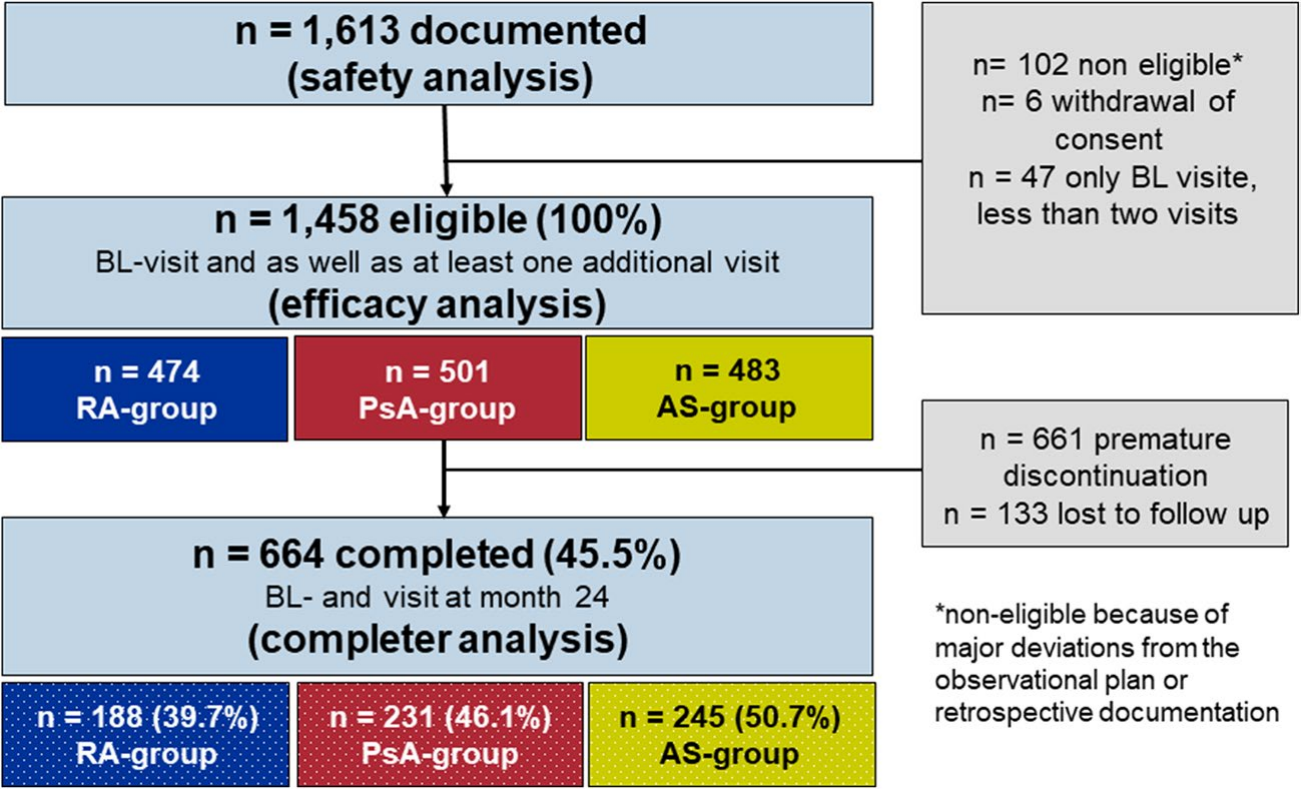
The flow chart shows the patient disposition and flow during the study. Of the 1613 documented patients (safety set), 1458 were eligible for the efficacy analysis (efficacy set), and 664 completed the study (completer set). *AS* ankylosing spondylitis, *BL* baseline, *PsA* psoriatic arthritis, *RA* rheumatoid arthritis

A total of 1458 patients had a baseline assessment as well as at least one additional visit and were eligible for final analysis (evaluable population): 474 (32.5%) with RA, 501 (34.4%) with PsA, and 483 (33.1%) with AS. The 24-month observational period was completed by 664 patients (45.5%). Details are shown in Fig. 1.

### Baseline characteristics of the evaluable population

At baseline (BL), mean age of patients was lowest in the patients with AS (43.6 ± 12.3 years) compared to those with PsA (50.3 ± 12.1 years) and RA (54.9 ± 13.4 years, Table 1). The proportion of females was lower in AS (33.5%) than in PsA (54.1%) and RA (72.8%).

**Table 1:** Baseline characteristics

	RA (*n* = 474)	PsA (*n* = 501)	AS (*n* = 483)
Demographics			
Age, years	54.9 ± 13.4	50.5 ± 12.1	43.6 ± 12.3
Range	19–82	18–83	18–73
Males, *n* (%)	129 (27.2)	230 (45.9)	321 (66.5)
Mean Body Mass Index, kg/m^2^	26.5 ± 4.9	28.1 ± 5.4	26.7 ± 5.5
Time since first diagnosis, years	10.4 ± 8.9	13.0 ± 11.5	9.8 ± 9.4
At least one concomitant disease	264 (55.7)	258 (51.5)	203 (42.0)
Patients with extra-articular manifestations	73 (15.4)	439 (87.8)	163 (33.9)
(Pre-)treatment status			
Biologic-naïve, *n* (%)	305 (64.3)	286 (57.1)	292 (60.5)
Patients currently on NSAIDs, coxibs, analgesics	258 (54.4)	333 (66.5)	416 (86.2)
Patients currently on basic therapy or immunosuppressants	407 (85.8)	322 (64.3)	133 (27.6)
Patients currently on syst. glucocorticoids	360 (75.9)	206 (41.1)	22 (4.6)
Employment status			
Full time, *n* (%)	154 (32.6)	225 (45.1)	298 (62.1)
Part time, *n* (%)	64 (13.1)	53 (10.6)	36 (7.5)
Unemployed, *n* (%)	24 (5.1)	50 (10.0)	52 (10.8)
Housewife/houseman, *n* (%)	51 (10.8)	30 (6.0)	16 (3.3)
Pupil/ student/ in apprenticeship, *n* (%)	9 (1.9)	15 (3.0)	11 (2.3)
Early pension, *n* (%)	29 (6.1)	46 (9.2)	21 (4.4)
Old-age pension, *n* (%)	128 (27.1)	63 (12.6)	29 (6.0)
Handicapped (unable to work in his/her Profession or work at all), *n* (%)	15 (3.2)	17 (3.4)	17 (3.5)

Values are mean ± standard deviations, or number of patients (percentages), respectively

In line with the age pattern, full-time employment was highest in patients with AS (62.1%) compared to those with PsA (45.1%) and RA (32.6%).

Mean disease duration since initial diagnosis was 10.4 years in RA, 13.0 years in PsA, and 9.8 years in AS. In terms of treatment history, patients without any prior biologics use were 64.3% in RA, 57.1% in PsA, and 60.5% in AS.

### Physician global assessment

**Fig. 2:**
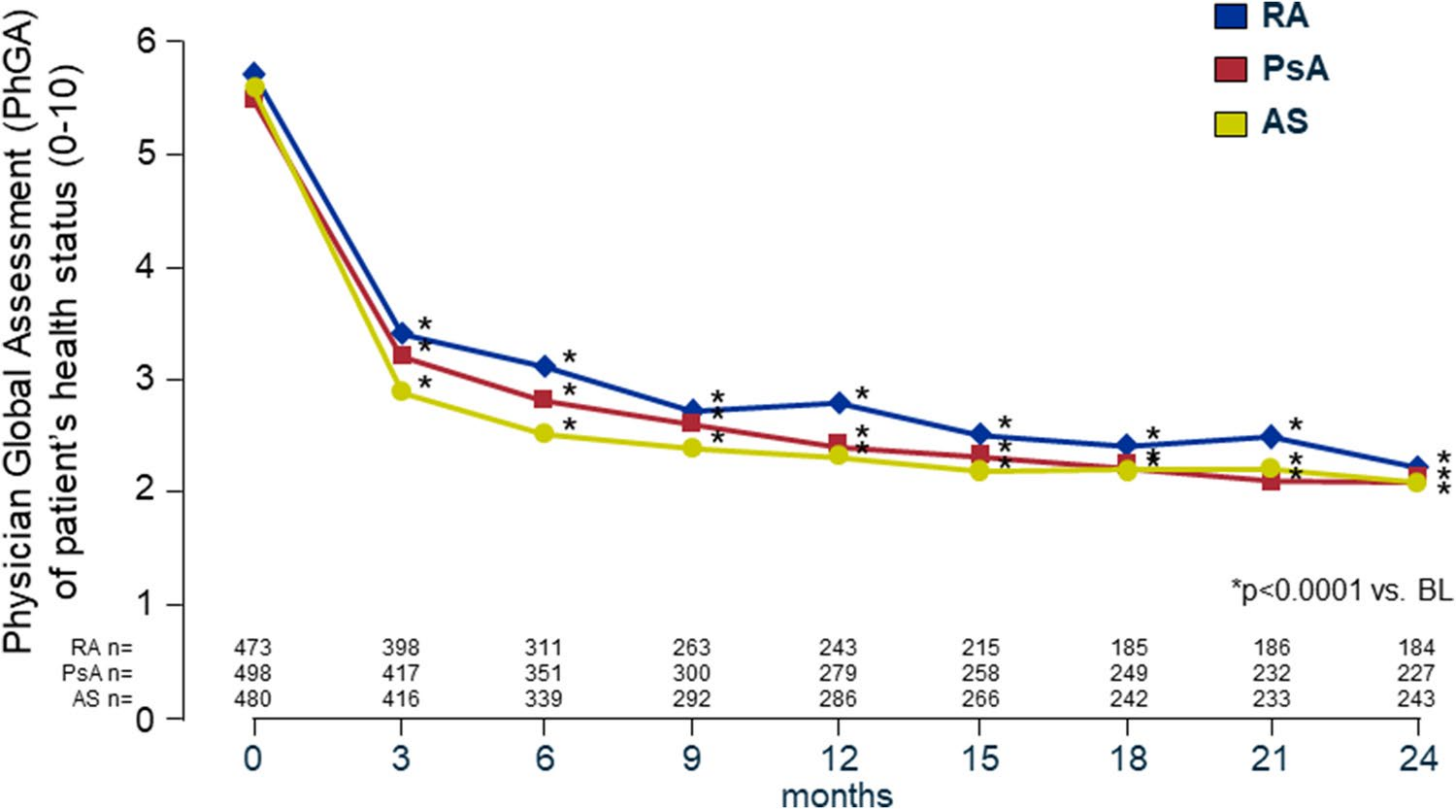
Physician Global Assessment (PhGA) of patient’s health status. The PhGA is a non-disease-specific evaluation of participants’ overall health status assessed on a 10-mm visual analogue scale (VAS) ranging from “0” (free of complaints) to “10” (strong discomfort). The closer the score is to 0, the better is the health status

In patients with RA the mean PhGA on the 10-point VAS improved significantly from 5.7 at BL to 3.4 (− 2.3) at month 3 and to 2.2 (− 3.5) at month 24, in patients with PsA from 5.5 at BL to 3.2 (− 2.3) at month 3, and to 2.1 (− 3.4) at month 24, and in patients with AS from 5.7 at BL to 2.9 (− 2.8) at month 3, and to 2.1 (− 3.6) at month 24 (*p* < 0.0001 vs. BL, each) (Fig. 2).

### Patient-reported outcomes

#### QoL by EQ VAS

**Fig. 3:**
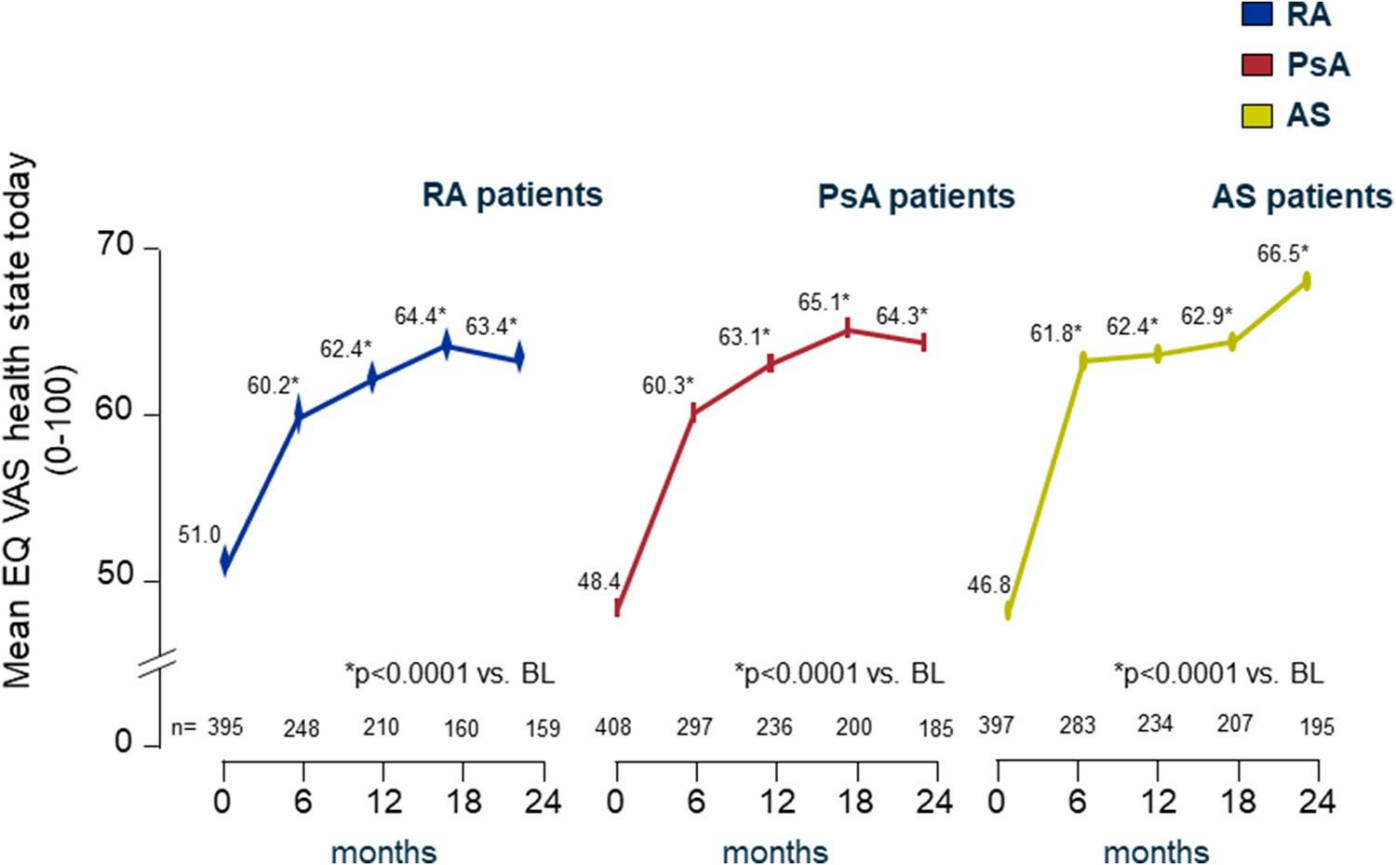
EQ visual analogue scale (EQ VAS) from 0 = ‘worst imaginable health state’ to 100 = ‘best imaginable health state’. *BL* baseline

Patients’ self-reported health state by EQ VAS improved in all indications, and the effect was maintained throughout the observation (Fig. 3).

The patients’ health state today (EQ VAS) improved significantly (*p* < 0.0001 vs. BL, each) from 51.0 at baseline (BL) to 63.4 (+ 12.4) (RA), from 48.4 to 64.3 (+ 15.9) (PsA), and from 46.8 to 66.5 (+ 19.7) (AS) during the course of treatment within 24 months.

The benefit was most pronounced within the first 6 months in all disease groups.

#### EQ-5D-3L

**Fig. 4:**
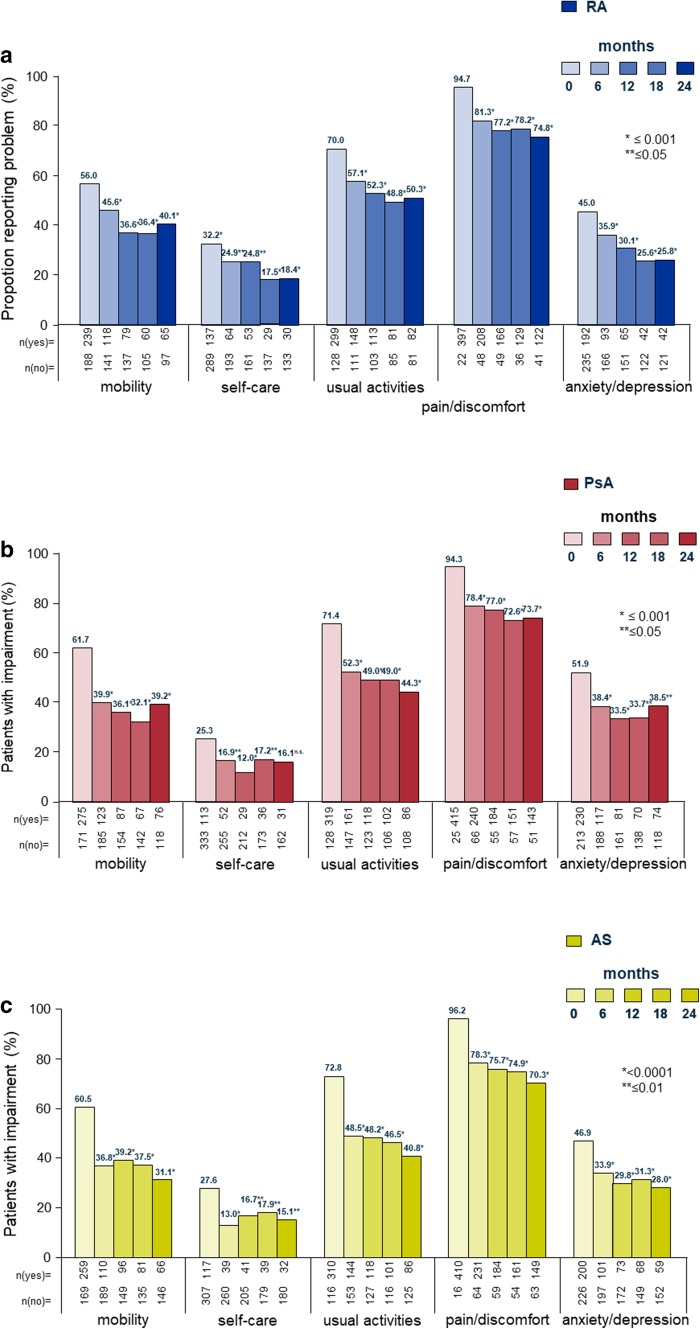
EQ-5D-3L: Euro-QoL descriptive system of health-related quality of life states consisting of five dimensions and three levels. The figure shows the proportion of patients reporting some or extreme problems, by visit

Patients most frequently reported impairments in the domain pain/discomfort, followed by usual activities, mobility, anxiety/depression, and self-care. In all disease groups, substantial improvements (all but one statistically significant) were noted during follow-up compared to BL. Effects occurred early and were maintained throughout the study (Fig. 4a–c).

#### Functional ability (FFbH)

**Fig. 5:**
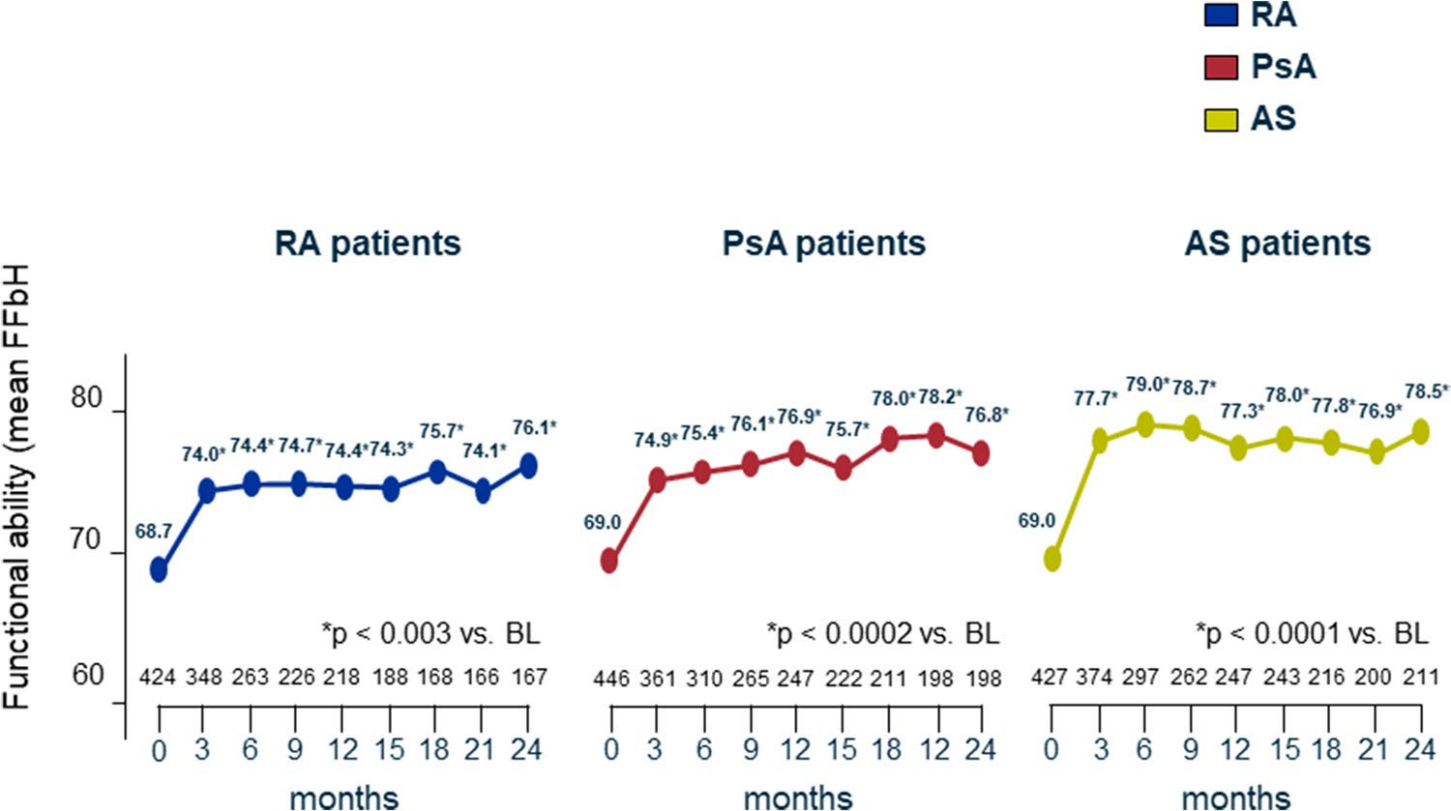
FFbH: Funktionsfragebogen Hannover. 0 represents minimal function, 100 optimal function

An improvement in functional ability by FFbH was also shown during the 24 months of therapy. The mean FFbH score increased significantly from baseline 68.7 to 74.0 (+ 5.3) at month 3 (*p* < 0.001) and 76.1(+ 7.4) at month 24 in patients with RA, from 69.0% to 74.9(+ 5.9) at month 3 (*p* < 0.0002) and 76.8(+ 7.8) at month 24 in patients with PsA, and from 69.0to 77.7(+ 5.9) at month 3 (*p* < 0.0001) and 78.5(+ 9.5) at month 24 in patients with AS (Fig. 5).

#### Functional assessment of chronic illness therapy: fatigue (FACIT-F)

**Fig. 6:**
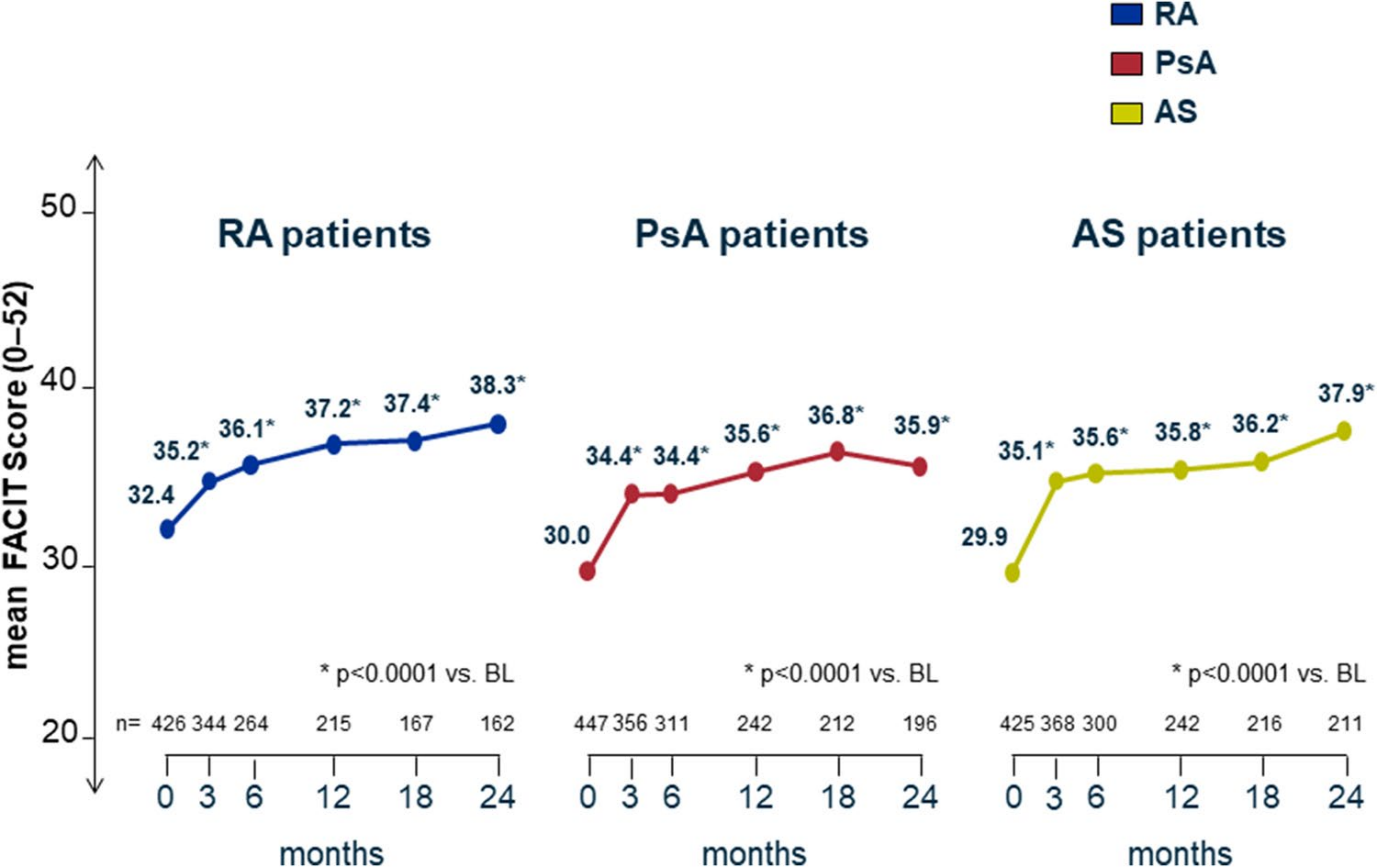
FACIT-Fatigue: functional assessment of chronic illness therapy-fatigue. On the FACIT-F, 0 points represent the worst and 52 the highest possible state with regard to fatigue

The mean FACIT-Fatigue score increased significantly (*p* < 0.0001) in patients with RA from baseline 32.4 to 35.8 (+ 3.4) at month 3 and to 38.3 (+ 5.9) points at month 24, in patients with PsA from 30.0 to 34.4 (+ 4.4) at month 3 and to 35.9 (+ 5.9) points at month 24, and in patients with AS from 29.9 (+ 5.2) to 35.1 at month 3 and to 37.9 (+ 8.0) points at month 24 (Fig. 6).

#### Safety

There were no previously unknown adverse drug effects detected in the study.

## Discussion

The present study is the first to report real-world data on PRO on the three main inflammatory rheumatic diseases RA, PsA, and AS for which GLM is approved. GLM administered in the 50 mg dose subcutaneously in monthly intervals was an effective treatment in patients with RA, PsA, and AS in a real-life setting in Germany. During the 2-year observation, substantial improvements, patient-reported quality of life, functional capacity, and fatigue were seen. The study complements the findings of various phase III controlled studies which were the basis for regulatory approval [19].

The mentioned randomised controlled studies reported limited health-related QoL data. In the RA GO-AFTER study, only FACIT-F and visual analogue scales for pain or overall disease activity were used, but no instrument for generic QoL assessment [29]. Also the pivotal RA studies GO-BEFORE and GO-FORWARD have not presented health-related QoL data [11, 30]. In AS GO-RAISE study, the Short-Form (SF-) 36 [31] was used and the physical and mental components showed clear improvement [13, 32]. In the PsA GO-REVEAL study, the physical component of the SF-36 was also improved [12].

The present study adds, for the first time, data on the EQ-5D-3L and  EQ VAS, over 2 years’ follow-up. Effects of GLM treatment on QoL, fatigue, and functional ability were substantial and were maintained over the full observation period.

The FFbH has been shown earlier to be a reliable and valid instrument for measuring functional disability in a German-speaking population with RA [24] In all groups in GO-NICE, scores increased in a clinically relevant manner by the end of the study. They were higher compared to a recent large observational study in RA with another biologic which used the FFbH as primary endpoint (mean score at 1 year was approximately 65 points) [33].

Fatigue has increasingly been recognized as an important clinical dimension by patients with rheumatic diseases [34]. In a recent international survey, RA patients identified pain, fatigue, and independence as the most important domains of disease activity that need to be improved to reflect remission [35]. For the assessment of fatigue, the FACIT-F scale is one of the most widely used multidimensional tools [36]. The average scores in the present study are in line with those reported in a study in 557 RA patients (28 to 32 points depending on DMARD or biologic treatment, with no significant differences) [37]. The positive treatment effect in GO-NICE is in line with a number of clinical trials in RA [37], in PsA [38], and in AS [39].

### Methodological considerations

PRO are intrinsically subjective and present a range of scientific and logistical challenges. For example, in surveys in the UK, researches and trial personnel have stated substantial inconsistencies in the level of assistance given to trial participants, the timing of PRO materials’ completion in relation to the clinical consultation, and the routine screening for avoidable missing data in order to maximise data quality/minimise risk of bias [40, 41].

This study has several strengths and limitations. Strengths include the relatively large cohort of prospectively enrolled and consecutive patients with one of the three index diagnoses which were mainly treated by rheumatologists. The initial responses to GLM treatment as well as overall outcomes match the effects seen in the phase III pivotal trials what externally validates the results.

As limitations, the relatively high rate of patients who were lost to follow-up must be considered (with no information on the outcomes on these patients). Similar rates as in GO-NICE have been reported from the US-American Corrona RA Registry, where almost half of patients discontinued bDMARD therapies within 24 months [42].

All patients came from Germany which limits the generalizability of the findings to patients in other health care settings and countries. A further important limitation is the lack of a control group of patients who did not receive GLM therapy. Clinical decisions of the treating physicians may assign selected patients to GLM as compared to other treatment options, what potentially may introduce allocation or channelling bias and confound the association between treatment and outcomes [43]. Physicians and patients willing to participate in non-interventional studies such as GO-NICE may be particularly motivated or interested in science and, therefore, also be subject to selection bias. A major limitation is the lack of data concerning concomitant drug use (csDMARD) during the follow-up period that could possibly interfere with the PROs.

## Conclusions

Golimumab (GLM) 50 mg SC monthly was an effective treatment in patients with RA, PsA, and AS in a real-life setting in Germany. During the 24-month observation, substantial improvement in patient-reported quality of life (EQ-5D-3L and EQ VAS), functional capacity (FFbH), and fatigue (FACIT-F) were seen in a very similar manner in all three diseases.

## Abbreviations

AS: Ankylosing spondylitis
EULAR: European League Against Rheumatism
FACIT-F: Functional Assessment of Chronic Illness Therapy Fatigue
FFbH: Functional ability
GLM: Golimumab
PRO: Patient-reported outcomes
PsA: Psoriatic arthritis
QoL: Quality of life
RA: Rheumatoid arthritis
SC: Subcutaneous
TNFi: Tumour necrosis factor inhibitors
